# Three-year Clinical Performance of a Universal Adhesive in Non-Carious Cervical Lesions

**DOI:** 10.3290/j.jad.b4186751

**Published:** 2023-06-30

**Authors:** Marleen Peumans, Stefanie Vandormael, Iris De Coster, Jan De Munck, Bart Van Meerbeek

**Affiliations:** a Full Professor, KU Leuven (University of Leuven), Department of Oral Health Sciences, BIOMAT & UZ Leuven (University Hospitals Leuven), Dentistry, Leuven, Belgium. Set up study design, wrote the manuscript.; b Master-after-Master Student in Restorative Dentistry, KU Leuven (University of Leuven), Department of Oral Health Sciences & UZ Leuven (University Hospitals Leuven), Dentistry, Leuven, Belgium. Organized patient 3-year recalls, calculated results, tables and figures, proofread the manuscript.; c Master-after-Master Student in Restorative Dentistry, KU Leuven (University of Leuven), Department of Oral Health Sciences & UZ Leuven (University Hospitals Leuven), Dentistry, Leuven, Belgium. Patient selection, performed placement of restorations, baseline and 1-year recall, proofread the manuscript.; d Post-Doctoral Research Fellow, KU Leuven (University of Leuven), Department of Oral Health Sciences, BIOMAT, Leuven, Belgium. Carried out the statistical analysis, proofread the manuscript.; e Full Professor, KU Leuven (University of Leuven), Department of Oral Health Sciences, BIOMAT & UZ Leuven (University Hospitals Leuven), Dentistry, Leuven, Belgium. Proofread the manuscript.

**Keywords:** randomized clinical trial, universal adhesive, application modes, non-carious cervical lesions, clinical effectiveness, bonding

## Abstract

**Purpose:**

The aim of this randomized controlled clinical trial was to evaluate the 3-year clinical performance of a universal adhesive (Clearfil Universal Bond Quick (CUBQ); Kuraray Noritake) when restoring non-carious cervical lesions (NCCLs) using two different application modes (etch-and-rinse vs self-etch with prior selective enamel etching).

**Materials and Methods:**

Fifty-one patients participated in this study. A total of 251 NCCLs (n = 251) were assigned to two groups: 1) CUBQ applied in etch-and-rinse mode (n = 122; CUBQ-ER) and 2) CUBQ applied in self-etch mode with prior selective etching of enamel with phosphoric acid (n = 129; CUPQ-SEE). The same resin composite, Clearfil Majesty ES-2 (Kuraray Noritake), was used for all restorations. The restorations were evaluated at baseline, 1 and 3 years using FDI criteria: marginal staining, fracture and retention, marginal adaptation, post-operative sensitivity and recurrence of caries. Statistical analysis was performed using a logistic regression model with generalized estimating equations (2-way GEE model).

**Results:**

The patient recall rate at 3 years was 90%. After 3 years, both groups presented an increase in the percentage of small but still clinically acceptable marginal defects (CUBQ-ER: 67%, CUBQ-SEE: 63.2%) and marginal staining (CUBQ-ER: 32.6%, CUBQ-SEE: 31.7%). The overall success rate was 82.6% and 83.8% for CUBQ-ER and CUBQ-SEE, respectively. In total, 38 restorations (19 CUBQ-ER, 19 CUBQ-SEE) failed because of loss of retention, fracture, severe marginal defect and/or marginal discoloration. A retention rate of 87.2% and 86.3% was recorded for CUBQ-ER and CUBQ-SEE, respectively. No significant difference was observed between the two bonding-mode groups for any of the evaluated parameters.

**Conclusion:**

After 3 years of clinical service, Clearfil Universal Bond Quick performed similarly in etch-and-rinse and self-etch modes with prior selective enamel etching.

Modern adhesives can be classified as etch-and-rinse (ER) or self-etch adhesives (SE).^62^ Etch-and-rinse adhesives are more technique sensitive, as there is a risk of collagen-fiber collapse and incomplete impregnation of the entire demineralized dentin. Self-etch adhesives do not require a separate etch-and-rinse phase, because they contain acidic monomers that simultaneously demineralize and infiltrate the dentin substrate. Therefore, these adhesives are promoted as a less time-consuming and less technique-sensitive alternative.

The latest generation of adhesives are being referred to as “universal” adhesives (UAs), which combine the primer with the adhesive resin to make the adhesive procedure even faster and less technique sensitive. These UAs can be used following either an ER or SE approach, or an SE approach with prior selective etching of enamel (SEE). The practicing dentist can determine which adhesive strategy to use on the basis of the actual clinical cavity conditions. In terms of enamel and dentin bonding, several investigations have revealed that universal adhesives exhibit equal or higher bonding effectiveness compared to the previous generation of 1-step SE adhesives.^36,51,53^ Most universal adhesives enable a chemical bond of functional monomers to hydroxyapatite, when used in SE bonding mode, which has been shown to contribute to the durability of the adhesive interface.^15,62^ Among the presently used functional monomers, 10-MDP has shown a strong and stable bond to dentin.^66,67^

Recently, some UAs have been marketed for immediate use, following a “no-wait” concept: the adhesive is applied, optionally using an active rubbing motion, and is light cured immediately upon application without any delay. Clearfil Universal Bond Quick (CUBQ, Kuraray Noritake; Tokyo, Japan) is a fluoride-releasing UA that is applied according to this “quick bonding” or “no-wait” concept. Following the manufacturer’s instructions, this universal adhesive can be readily light cured upon application, as there is no need for longer interaction with tooth tissue or extra time for the solvent to evaporate prior to light curing. According to the manufacturer, the quick bonding is made possible by its lower 2-hydroxyethyl methacrylate (HEMA) content (2.5% vs 10% in conventional UAs) and higher purity of the functional monomer 10-MDP, as well as the new acrylamide monomer technology. This acrylamide monomer has a higher hydrophilic potential than HEMA, which endows it with good wettability to tooth structure.^17,60^ Given the higher wettability of this monomer, the manufacturer insists upon immediate air drying after application of the adhesive. If done, it could be advantageous for good adhesion, especially in the cervical region, because the shorter manipulation time could avoid irritation from adhesion-reducing factors such as bleeding from the gingiva, gingival crevicular fluids and moisture in the oral cavity. Additionally, the short application time is clinically appealing for the clinician. This acrylamide monomer also shows a higher degree of polymerization than HEMA, reducing water absorption and thereby contributing to bond durability.^29^ Finally, CUBQ includes a new integrated photoinitiator chemistry, also used in Clearfil SE Bond 2 (Kuraray Noritake), which may provide more free radicals and lead to higher monomer conversion rates.^48^

Several in-vitro studies evaluating the bonding effectiveness of CUBQ have demonstrated an adequate bond strength to dentin and enamel, quite similar to that of other UAs, and without significant differences between ER and SE modes.^1-4,6,12,23,25,65^ In some in-vitro studies, however, the dentin bond strength in the SE mode was found to be more stable with time.^2,3,23,25^ Similarly, some authors observed less nanoleakage when CUBQ was bonded to dentin in SE mode compared to ER mode.^6,11^ During microscopic observation, CUBQ also showed a firm, tight adhesive interface for both the ER and SE modes, with an adhesive layer thickness of around 10 µm.^3,50^

Despite the importance of in-vitro studies attempting to predict the performance of biomaterials, additional clinical studies are essential to evaluate the clinical performance of this relatively new adhesive system, as clinical trials remain the best way to collect scientific evidence on the clinical effectiveness of an adhesive. At present, to the extent of the authors’ knowledge, there is only 1 clinical trial available in the literature which evaluated the bonding effectiveness of CUBQ in SE, ER and SEE mode in non-carious cervical lesions (NCCLs) after 24 months.^38^ A 100% success rate was recorded for the SEE and ER mode, while 13% of the SE restorations failed due to loss of retention. Indeed, NCCLs are the best lesions for clinically testing the bonding efficacy of an adhesive, because they do not provide any (or only minimal) macroretention.^40,62^ In addition, the bonding effectiveness to both dentin and enamel can be tested because the largest part of the tooth’s bonding surface consists of dentin, whereas a border of enamel can be found on the incisal side of the restoration. Moreover, these lesions are highly prevalent in our population and are becoming increasingly common as an aging population retains its teeth longer.^56,63^ Direct restorative treatment is indicated in any of following situations: 1) the structural integrity of the tooth is threatened, 2) the exposed dentin is hypersensitive, 3) pulp exposition is likely, 4) the patient has an esthetic complaint or 5) tooth-shape modification is necessary to increase the retention of a partial denture.^43^

The aim of the present study was to evaluate the 3-year clinical effectiveness of CUBQ in NCCLs in a self-etch mode with prior selective etching of enamel with phosphoric acid and in an etch-and-rinse mode. The null hypotheses was that there is no difference in clinical effectiveness between the two application modes.

## Materials and Methods

In this clinical trial, the bonding effectiveness of Clearfil Universal Bond Quick was tested in NCCLs following two different application modes: etch-and-rinse (ER) and self-etch mode with prior selective etching of enamel with 35% phosphoric acid (SEE). As a restorative material, Clearfil Majesty ES-2 (Kuraray Noritake) was used for all NCCL restorations. The composition and application procedure of the different adhesive materials are presented in Table 1.

**Table 1:** Composition and application procedure of the materials used

Materials	Manufacturer	pH	Composition	Application procedure
K-Etchant Syringe	Kuraray Noritake; Tokyo, Japan	1.8	Phosphoric acid (35-45%)	SE with selective etching of enamel: Apply K-etchant syringe to the beveled enamel for 10 s, thorough water rinsing (> 10 s), gentle air drying. E&R: Apply K-etchant syringe to the entire cavity (enamel and dentin) for 10 s, thorough water rinsing (> 10 s), gentle air drying
Clearfil Universal Bond Quick (C-UBQ)	Kuraray Noritake	2.3	Bis-GMA (10-25%), HEMA (2.5-10%), 10-MDP, hydrophilic amide monomer, colloidal silica, silane coupling agent, sodium fluoride, camphorquinone, ethanol (10-25%), water	Using bottle adhesive: Dispense the necessary amount of bond into a well of the dispensing dish immediately before application. Use the light-blocking plate to avoid exposing the material to an operating light or ambient light; use within 7 min after dispensing. Apply in rubbing motion to the entire cavity wall, no waiting time is required. Mild air blowing (≥5 s) until the adhesive no longer moves. Use a vacuum aspirator to prevent the adhesive from scattering. Light cure (10 s) (Demi Plus, Kerr, Light intensity 1100 mW/cm^2^)
Clearfil Majesty ES-2	Kuraray Noritake		Organic matrix: bis-GMA, hydrophobic aromatic dimethacrylate, camphorquinone. Inorganic filler (78 wt.%): silanated barium glass filler, pre-polymerized organic filler including nanofiller Initiators, accelerators, pigments.	Apply Clearfil Majesty ES-2, light cure, finish and polish

Bis-GMA: bisphenol-A-glycidyl-dimethacrylate; HEMA: 2-hydroxyethyl methacrylate; 10-MDP: 10-methacryloyloxydecyl dihydrogenphosphate.

### Patient and Lesion Selection

The clinical trial protocol was approved by the Commission for Medical Ethics of UZ Leuven (University Hospitals Leuven, project B322201731313).

Study subjects were non-hospitalized patients at the University Hospital (UZ Leuven) who needed dental treatment of NCCLs. Reasons for treatment included tooth sensitivity, prevention of further tooth wear and/or esthetic complaints. Patients with a complex medical history, severe or chronic periodontitis, high caries risk or severe bruxism were excluded from the study. Prior to their enrollment, patients were informed of the nature and objectives of the clinical study before signing a written consent.

A total of 51 patients were included and 251 restorations were placed. Of the 251 restorations, 122 restorations were placed with the adhesive used in an etch-and-rinse mode (CUBQ-ER). For the other 129 restorations, the adhesive was used in a self-etch mode with prior selective etching of enamel with phosphoric acid (CUBQ-SEE).

Tables 2 and 3 show the baseline data regarding patients and lesions included in the study.

**Table 2:** Baseline data regarding the patients enrolled in the study

	Number of patients
	Total
Total number of patients	51
**Gender distribution**	
Female	31
Male	20
**Age distribution (years)**	
31-40	6
41-50	10
51-60	10
61-70	13
71-81	12
**Smoking habits**	
Smoker	5
Non-smoker	46
**Oral hygiene**	
Good	43
Slight gingivitis	7
Severe gingivitis	1

**Table 3:** Baseline data regarding the lesions included in the study

Characteristics of the treated cervical lesions	Evaluation method	Number of restorations		
		CUBQ-SESE	CUBQ-E&R	Total
Untreated/previously treated lesion				
Untreated non-carious lesion		102	97	199
Carious lesion		2	0	2
Old restoration with caries recurrence		0	0	0
Old restoration without caries recurrence		16	17	33
Arrested cares		0	1	1
Traumatic lesion		9	7	16
**Vitality**	**Thermal sensitivity test**			
Vital		118	115	233
Non vital – retracted pulp		9	7	16
Non-vital (endodontic treatment)		2	0	2
**Pre-operative sensitivity index**	**Air stream and moving a probe over the lesion**			
Normal sensitivity		109	99	208
Increased sensitivity		20	23	43
**Shape (of the untreated lesion)**	**Visual/tactile (probe)**			
Sharply defined, wedge-shaped		80	64	144
Rounder, saucer-shaped		49	58	107
**Depth of lesion**	**Periodontal probe**			
Shallow (< 1 mm)		74	70	144
Deep (> 1 mm)		55	52	107
**Cervico-incisal height of lesion**	**Periodontal probe**			
< 1.5 mm		18	15	33
1.5 – 2.5 mm		41	32	73
> 2.5 mm		70	75	145
**Degree of sclerosis**	**Visual 52**			
No sclerosis	Non-opaque dentin	24	24	48
Slightly sclerotic	Opake dentin	54	47	101
Intermediately sclerotic	Yellow dentin	39	47	86
Strongly sclerotic	Transparent dentin	12	4	16
**Presence of wear facets**	**Visual (after air drying)**			
No wear facets		67	60	127
Wear facets		62	62	124
**Presence of antagonist**				
Antagonist present		122	112	234
No antagonist present		7	10	17
**Isolation index**				
Rubber-dam		116	89	214
Metal matrixband + cotton rolls		0	1	1
Retraction cord + cotton rolls		13	23	36
**Tooth distribution**				
Maxillary incisors		7	11	18
Mandibular incisors		10	9	19
Maxillary canines		17	11	28
Mandibular canines		11	10	21
Maxillary premolars		21	31	52
Mandibular premolars		51	37	88
Maxillary molars		8	7	15
Mandibular molars		4	6	10

### Sample Size Consideration and Randomization

Two hundred fifty-one (251) Class-V restorations were placed in 51 patients following a paired-tooth design. For each patient, half of the lesions were treated with CUBQ-ER and the other half with CUBQ-SEE. A randomization procedure was followed to assign the teeth to be restored following an adhesive technique (using randomization tables), whereby the first randomly selected adhesive technique was used to restore the tooth with the lowest tooth number (according to the FDI system), and the alternative adhesive technique was used for the tooth with the second lowest tooth number. This method was used for every other tooth requiring a cervical restoration. In case of an uneven number of restorations placed in one patient, the inequality of number of teeth restored with one adhesive technique was adjusted by restoring one more lesion with the other adhesive technique in the next patient presenting with an unequal number of cervical lesions (again according to the respective randomization tables).

### Restorative Procedure

Four specially-instructed dentists from the University Dental School, with 1-3 years of clinical experience in restorative dentistry, performed all restorative procedures.

Local anesthesia was given if needed to prevent patient discomfort during restorative procedures. The teeth to be restored were first cleaned with a pumice-water slurry using a rubber cup to remove the salivary pellicle and any remaining dental plaque. The dentin walls of the lesions were gently and superficially roughened using a coarse diamond bur prior to the conditioning and bonding procedure. During this step, superficial caries was removed if present. No lining material was applied. All sharp enamel and dentin margins were rounded. A short 1- to 2-mm enamel bevel was prepared. Almost all restorative procedures were carried out under rubber-dam isolation with a gingival clamp (Brinker Tissue set, Hygienic, Coltene-Whaledent; Altstätten, Switzerland) to retract the gingival tissue (n = 214). If placement of the gingival retraction clamp was not possible, other methods of isolation were used, eg, a metal matrixband (Automatrix, Dentsply Sirona; Konstanz, Germany) (n = 1) or a retraction cord in combination with cotton rolls (n = 36). The adhesive materials were applied following the manufacturer’s instructions (Table 1). Next, the restorative composite (Clearfil Majesty ES-2) was applied incrementally. Each layer was cured for 20 s with an LED light-curing unit (Demi, Kerr; Orange, CA, USA) with a minimal light output of 1100 mW/cm^2^. The cervical lesions were restored to their natural contour. Final contouring and finishing of the restorations were performed during the same appointment, using a microfine (40 µm) pointed diamond bur at high speed under water cooling (Komet FG 8862.314.012, Gebr. Brasseler; Lemgo, Germany) to remove the excess composite on the surface and at the margins. Next, the composite surface was prepolished and polished with rubber polishing wheels (dry and with air cooling) (Twist Dia prepolisher and polisher, Kuraray Noritake).

### Evaluation Procedure

Clinical evaluation took place at baseline (approximately 1 week after placement of the restorations) by two calibrated investigators (MP, BVM) who were fully blinded to the kind of adhesive technique that was used. Any marginal adaptation defects (overhangs) were still corrected at this stage. Subsequently, all patients were subjected to a recall schedule with controls at 1 and 3 years. For clinical evaluation of the restorations, the FDI clinical criteria and scoring system were employed.^20-22,34^ The clinical effectiveness was recorded in terms of marginal staining, fracture and retention, marginal adaptation, post-operative sensitivity, and recurrence of caries/erosion/abfractions (Table 4). Separate evaluation of enamel and dentin margins expanded the scope of the original FDI criteria. The criteria of marginal staining and marginal adaptation were modified as follows: marginal staining on the enamel side and/or dentin side; marginal adaptation on the enamel side and/or dentin side. Clinical photographs were made pre-operatively (situation before, after isolation), at baseline and at 1 and 3 years. Any discrepancy in evaluation between the two evaluators was immediately resolved at chair side.

**Table 4:** Word Dental Federation (FDI) criteria used for clinical evaluation

	Esthetic properties	Functional properties		Biological properties	
	Marginal staining	Fractures and retention	Marginal adaptation	Postoperative sensitivity	Recurrence of caries, erosion, abfraction
Evaluation criteria	Visually (after air drying the tooth)	Visually (after air drying the tooth) and tactilely using a sharp probe	Tactilely with special probes with tip diameters of 150 µm and 250 µm. The depth of the gap should be at least the same size.	Applying a stream of compressed air for 3 s at distance of 2-3 cm (while shielding the adjacent teeth with fingers); moving a probe over the lesion	Visually and tactilely using a probe (after air drying the tooth).
Clinically excellent/very good	No marginal staining	Restoration retained, no fractures/ cracks	Harmonious outline, no gaps, no discoloration	4.1. No hypersensitivity	5.1. No secondary or primary caries
Clinically good (after polishing probably very good)	Minor marginal staining, easily removable by polishing	Small hairline cracks	3.2.1. Marginal gap (50 µm) (1. at enamel/ 2. at dentin side) 3.2.2. Small marginal fracture removable by polishing. (1. at enamel/ 2. at dentin side) 3.2.3. Slight ditching, slight step/flashes, minor irregularities (1. at enamel/ 2. at dentin side)	4.2. Low hypersensitivity for a limited period of time	5.2. Small and localized: 1. Demineralization 2. Erosion 3. Abfraction
Clinically sufficient/satisfactory (Minor shortcomings, no unacceptable effects but not adjustable without damage to the tooth)	Moderate marginal staining, not aesthetically unacceptable	Two or more or larger hairline cracks and/or chipping (not affecting the marginal integrity).	3.3.1. Gap < 250 µm not removable (1. at enamel/ 2. at dentin side) 3.3.2. Several small enamel or dentin fractures (1. at enamel/ 2. at dentin side) 3.3.3. Major irregularities, ditching of flash, steps (1. at enamel/ 2. at dentin side)	4.3.1. Premature/slightly more intense 4.3.2. Delayed/weak sensitivity; no subjective complaints, no treatment needed	5.3. Larger areas of 1. Demineralization 2. Erosion 3. Abfraction/abrasion, Dentin not exposed/ Only preventive measures necessary
Clinically unsatisfactory (but reparable)	Pronounced marginal staining; major intervention necessary for improvement	Chipping fractures which can damage marginal quality; bulk fractures with or without partial loss (less than half of the restoration)	3.4.1. Gap > 250 µm or dentin/ base exposed. (1. at enamel/ 2. at dentin side) 3.4.2. Severe ditching or marginal fractures (1. at enamel/ 2. at dentin side) 3.4.3. Larger irregularities or steps (1. at enamel/ 2. at dentin side)	4.4.1. Premature/very intense 4.4.2. Extremely delayed/ weak with subjective complaints. 4.4.3. Negative sensitivity: Intervention necessary but not replacement	5.4.1. Caries and cavitation and suspected undermining caries 8.4.2. Erosion in dentin 8.4.3. Abrasion/ Abfraction in dentin. Localized and accessible, can be repaired

### Statistical Analysis

The 3-year clinical effectiveness of both CUBQ-ER and CUBQ-SEE were compared for the different key parameters, as described in the evaluation criteria. A logistic regression model with generalized estimator equations (2-way GEE model), using a compound symmetry structure for the working correlation matrix, was used to account for the clustered data (multiple lesions per patient). The analyses were performed using a statistical software package (Geepack library and R 2.13.2, R Foundation for statistical Computing; Vienna, Austria). Odds ratio and 95% confidence intervals were computed.

## Results

The patient recall rate at 3 years was 90%: 46 out of 51 patients were examined. Three patients chose not to return during the COVID pandemic. One patient moved abroad, and one patient was no longer reachable by phone or e-mail. Because five patients did not return at the 3-year recall, there was a dropout of 23 restorations (12 CUBQ-ER, 11 CUBQ-SEE). Prior to the 1-year recall, one patient received a tooth extraction for periodontal reasons and another patient received a crown, resulting in two additional restoration dropouts. In total, 109 of the 122 CUBQ-ER restorations and 117 of the 129 CUBQ-SEE restorations were evaluated at the 3-year recall.

**Fig 1:**
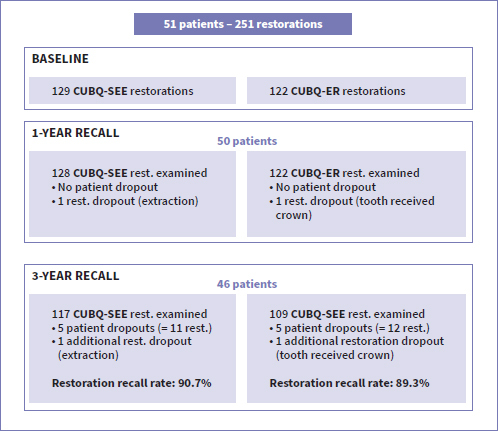
CONSORT Flow Diagram. CUBQ-ER: Clearfil Universal Bond Quick is used in an etch-and-rinse mode; CUBQ-SEE: Clearfil Universal Bond Quick is used in a self-etch mode with prior selective etching of enamel with phosphoric acid; rest.: restoration.

Figure 1 shows the number of patients and restoration dropouts at each recall. The results of the evaluation criteria at each recall are presented in Table 5.

**Table 5:** Evaluation results in percentage at each evaluation period

Evaluation criterion	FDI score	Evaluation period					
		Baseline		1 year		3 years	
		CUBQ-SEE	CUBQ-ER	CUBQ-SEE	CUBQ-ER	CUBQ-SEE	CUBQ-ER
**Marginal staining**	**1**						
No marginal staining	1.1	100	100	81.9	85.8	67.3	67.4
Superficial marginal staining	1.2 and 1.3	0	0	17.2	14.2	31.7	32.6
On enamel side		0	0	7.8	3.5	10.9	15.8
On dentin side		0	0	7.8	7.1	14.9	11.6
On enamel and dentin side		0	0	1.7	3.5	5.9	5.3
Severe marginal staining	1.4 and 1.5	0	0	0.9	0	1	0
On enamel side		0	0	0	0	0	0
On dentin side		0	0	0.9	0	1	0
On enamel and dentin side		0	0	0	0	0	0
**Fracture and retention**	2						
No fracture	2.1	100	99.2	89.8	92.6	83.8	84.4
Acceptable fracture	2.2 and 2.3	0	0	0	0	0	0
Unacceptable fracture (reparable)	2.4	0	0	0.8	0.8	2.5	2.8
Partial/complete loss of restoration	2.5 or 3.5	0	0.8	9.4	6.6	13.7	12.8
**Marginal adaptation**	3						
No marginal defect	3.1	93	91	27.3	28.9	22.2	16.5
Small marginal defect	3.2 and 3.3	7	8.2	62.5	62.8	63.2	67
On enamel side	3.2.1 and 3.3.1	0.8	1.6	16.4	20.7	14.5	16.5
On dentin side	3.2.2 and 3.3.2	6.2	6.6	14.8	14	11.1	13.8
On enamel and dentin side	3.2.3 and 3.3.3	0	0	30.5	24	31.6	30.3
Severe marginal defect	3.4	0	0	0.8	0.8	0.9	2.7
On enamel side	3.4.1	0	0	0	0	0	0
On dentin side	3.4.2	0	0	0	0	0.9	1.8
On enamel and dentin side	3.4.3	0	0	0.8	0.8	0	0.9
**No post-operative sensitivity**	4.1	98.4	98.3	100	99.1	100	100
**Caries/erosion/abrasion**	5.1	0	0	0	0	0	0
**Failure**		0	0.8	10.9	8.3	16.2	17.4

CUBQ-SEE: Clearfil Universal Bond Quick used in self-etch mode with prior selective enamel etching; CUBQ-ER = Clearfil Universal Bond Quick used in etch&rinse mode.

### Success Rate

The overall success rate at the 3-year recall was 82.6% and 83.8% for CUBQ-ER and CUBQ-SEE, respectively. In total, 38 restorations (19 CUBQ-ER, 19 CUBQ-SEE) failed for one of the following reasons: loss of retention (16 CUBQ-ER, 14 CUBQ-SEE), fracture (2 CUBQ-ER, 2 CUBQ-SEE), severe marginal defect (3 CUBQ-ER, 1 CUBQ-SEE) and/or discoloration (1 CUBQ-SEE). No significant difference in success rate was recorded between the two application modes (p > 0.05) (Table 6).

**Table 6:** Statistical analysis of the different parameters for clinical success of CUBQ-SEE versus CUBQ-ER (2-way GEE analysis)

	FDI scores	odds_ratio	odds_Lower	odds_Upper	p-value
No marginal staining	1.1	1.04998	0.59354	1.85740	0.8669
Superficial marginal staining	1.2+1.3	0.9796	0.55311	1.7351	0.9438
Superficial marginal staining, enamel side		1.56068	0.78288	3.11121	0.2060
Superficial marginal staining, dentin side		0.73649	0.29531	1.83679	0.5118
Severe marginal staining	1.4+1.5	0.00000	0.00000	0.00000	
Severe marginal staining, dentin side		Inf	Inf	Inf	
No fracture	2.1	1.06585	0.49220	2.30805	0.8715
Restoration loss	2.5 or 3.5	0.90840	0.38701	2.13220	0.8253
No marginal defect	3.1	0.66684	0.35974	1.23609	0.1981
Clinically acceptable marginal adaptation	3.2+ 3.3	0.88201	0.40309	1.92997	0.7533
Small marginal defect on enamel side	3.2.1+3.3.1	1.11946	0.58756	2.13290	0.7315
Small marginal defect on dentin side	3.2.2+3.3.2	1.29413	0.68459	2.44639	0.4274
Small marginal defect on enamel and dentin side	3.2.3+3.3.3	0.9843	0.5311	1.8240	0.9598
Clinically unacceptable marginal adaptation	3.4+3.5	4.4384	0.7824	25.178	0.0924
Severe marginal defect on enamel side	3.4.1	Inf	Inf	Inf	
Severe marginal defect on dentin side	3.4.2	2.17788	0.53500	8.86582	0.2772
Severe marginal defect on enamel and dentin side	3.4.3	4.43843	0.78240	25.17841	0.0924
Absence of post-operative sensitivity	4.1				
Absence of caries/erosion/abrasion	5.1				
Failure		1.06831	0.51959	2.19653	0.8574

Regarding the parameters “marginal defects” and “marginal staining”, only the results for retained restorations were compared. odds_ratio: ratio of the odds of the event for CUBQ-SEE compared to the odds for CUBQ-ER; odds_Lower: lower limit of the 95% confidence interval; odds_Upper: upper limit of the 95% confidence interval.

### Fracture and Retention Rate

At the 3-year recall, two CUBQ-ER restorations and two CUBQ-SEE restorations showed an unacceptable severe chip fracture affecting marginal integrity. These restorations were reparable.

Thirty restorations (14 CUBQ-ER, 16 CUBQ-SEE) were lost, resulting in a retention rate of 87.2% in the CUBQ-ER group and 86.3% in the CUBQ-SEE group.

At baseline, 1-year and 3-year recall, the CUBQ-ER group lost 1, 8, and 14 restorations, respectively. In the CUBQ-SEE group, 12 and 16 restorations were lost at the 1-year and 3-year recall, respectively.

### Marginal Adaptation

**Fig 2:**
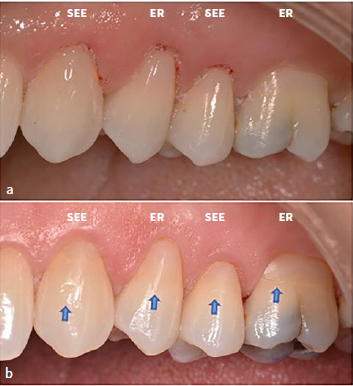
a. Baseline situation of NCCL composite restorations on 4 maxillary teeth (canine to first molar); b. 3-year recall: all restorations showed a small but still clinically acceptable marginal defect at the incisal enamel side (blue arrows).

The percentage of restorations with a perfect marginal adaptation decreased from baseline to the 1-year recall and decreased further at the 3-year recall (16.5% CUBQ-ER, 22.2% CUBQ-SEE). Most of the marginal defects were clinically acceptable (Fig 2).

At the 3-year recall, these marginal defects were located on the incisal enamel side (16.5% CUBQ-ER, 14.5% CUBQ-SEE), the cervical dentin side (13.8% CUBQ-ER, 11.1% CUBQ-SEE), or the incisal enamel side and the cervical dentin side (30.3% CUBQ-ER, 31.6% CUBQ-SEE).

**Fig 3:**
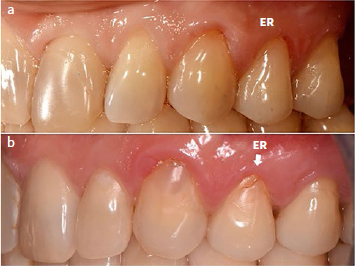
a. Baseline situation of NCCL composite restoration on tooth 24 (CUBQ-ER) b. 3-year recall: the restoration showed a fracture of the composite at the cervical margin resulting in a severe marginal defect at the cervical dentin side (white arrow). Repair of the restoration was needed.

Three CUBQ-ER and one CUBQ-SEE restorations showed an unacceptable severe marginal defect on the dentin side (1.8% CUBQ-ER, 0.9% CUBQ-SEE) (Fig 3) or on the enamel and dentin side (CUBQ-ER: 0.9%).

### Marginal Staining

Regarding marginal discoloration, a decrease was also observed in the percentage of restorations without marginal staining from baseline to the 3-year recall (67.4% CUBQ-ER, 67.3% CUBQ-SEE).

**Fig 4:**
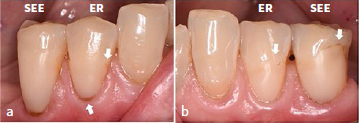
a. and b. 3-year follow up of the NCCL restorations showing superficial marginal discoloration on the enamel side and/or dentin side (white arrows).

Clinically acceptable marginal discoloration was detected either on the incisal enamel side (15.8% CUBQ-ER, 10.9% CUBQ-SEE), the cervical dentin side (11.6% CUBQ-ER, 14.9% CUBQ-SEE) or the incisal enamel side and cervical dentin side (5.3% CUBQ-ER, 5.9% CUBQ-SEE) (Fig 4). Only one CUBQ-SEE restoration showed unacceptable marginal discoloration on the cervical dentin side after 3 years of clinical service.

### Postoperative Sensitivity and Recurrence of Caries, Erosion, Abrasion, and Abfractions

Very few restored teeth (2 CUBQ-ER, 2 CUBQ-SEE) showed minor sensitivity at baseline. At the 3-year recall, sensitivity was no longer reported in either group. In addition, none of the restored teeth showed caries, erosion, abrasion or abfractions along the restoration margins at the 3-year recall.

No significant difference was recorded between the two groups (ER and SEE) for the above-mentioned parameters (p > 0.05). The results of the statistical analysis of the different parameters for clinical success (2-way GEE analysis) are presented in Table 6.

### Secondary Parameters

The influence of secondary parameters (operator, shape, depth and cervico-incisal height of the lesion, degree of sclerosis, presence of wear facets, presence of antagonist) on the failure rate of the restorations was statistically analyzed (2-way GEE model). The results are shown in Tables 7 and 8. No significant difference was noticed for shape, depth and cervico-incisal height of the lesion, presence of wear facets and antagonist, or isolation method. Only for sclerosis was a significant difference recorded, namely, for the interaction between application method and degree of sclerosis (p < 0.0001). Specifically, in the group without any sclerosis, the number of failures was significantly lower than in the other three groups (slightly sclerotic dentin, intermediately sclerotic dentin, strongly sclerotic dentin).

**Table 7a:** Relationship between secondary parameters of NCCLs and the number of failures at the 3-year recall (2-way GEE statistical analysis)

	CUBQ-SEE failed	CUBQ-ER failed	Total non-failed
**Shape of the lesion**			
Wedge shaped	10	5	120
Saucer shaped	6	9	76
**Depth of the lesion**			
<1 mm	9	8	108
≥1 mm	7	6	88
**Cervico-incisal height**			
<1.5 mm	3	0	28
1.5-2.5 mm	4	2	60
>2.5 mm	9	12	108
**Degree of sclerosis**			
No sclerosis	1	0	34
Slightly sclerotic	6	5	86
Intermediately sclerotic	7	9	63
Strongly sclerotic	2	0	13
**Wear facets**			
No wear facet	7	3	100
Wear facet	9	11	96
**Antagonist**			
Present	14	13	182
Absent	2	1	14
**Isolation**			
Rubber-dam	15	10	170
Metal matrix band + cotton rolls	0	0	1
Retraction cord + cotton rolls	1	4	25

**Table 7b:** Relationship between secondary parameters of NCCLs and the number of failures at the 3-year recall (2-way GEE statistical analysis)

2-way GEE analysis	p-value
Shape of the lesion	0.13
Baseline group	0.69
Shape : baseline group	0.19
Depth of the lesion	0.53
Baseline group	0.81
Depth : baseline group	0.71
Cervico-incisal height of lesion	0.47
Baseline group	0.71
Cervico-incisal height : baseline group	>0.9999
Degree of sclerosis	0.10
Baseline group	0.72
Sclerosis : baseline group	<0.0001***
Wear facets	0.14
Baseline group	0.76
Wear facet : baseline group	0.26
Presence of antagonist	0.43
Baseline group	0.79
Antagonist : baseline group	0.29
Isolation	0.52
Baseline group	0.76
Isolation : baseline group	0.26

**Table 8:** Relationship between operator and number of failures at the 3-year recall (2-way GEE statistical analysis)

	Failed restorations per operator							
	Operator 1		Operator 2		Operator 3		Operator 4	
No failure	38		20		40		90	
Failure	13		3		10		12	
	Failed restorations per operator per group							
	Operator 1		Operator 2		Operator 3		Operator 4	
	CUBQ-SEE	CUBQ-ER	CUBQ-SEE	CUBQ-ER	CUBQ-SEE	CUBQ-ER	CUBQ-SEE	CUBQ-ER
No failure	21	17	11	9	20	20	46	44
Failure	7	6	1	2	5	5	6	6
2-GEE analysis	Df	X2	p-value					
Operator	3	4.05	0.26					
Group	1	0.05	0.82					
Operator-Group	3	2.03	0.57					

Regarding the influence of the operator, operator 4, who placed the most restorations, showed the lowest number of failures (12/90), while operator 1 showed the highest number of failures (13/38) (Table 8). Nevertheless, the difference between the four operators was not significant (p=0.26). In addition, the number of failed restorations in each group (CUBQ-ER, CUBQ-SEE) was quite similar across operators (p=0.57).

## Discussion

The present clinical trial evaluated the bonding efficiency of a universal adhesive with a “quick bonding” concept (CUBQ) in NCCLs over a 3-year period. To the extent of the authors’ knowledge, this is the second clinical trial studying the bonding efficacy of this adhesive. According to the manufacturer’s instructions, the adhesive must be rubbed onto the dentin and enamel surface and, immediately after application, air thinned and light polymerized. The influence of application time of CUBQ on the bonding effectiveness to dentin and enamel has previously been tested in several in-vitro studies, showing varying results.^1,4,46,47^ On the one hand, several studies found no influence of the application time (application + immediate light curing or application vs light curing after 10-20 s) on the bond strength to enamel and dentin for both the ER and SE application modes.^37,46,47^ On the other hand, other in-vitro studies reported an increased bond strength to dentin after active application of 10-20 s.^1,4^ Atalay and Meral^4^ reported that the increase in dentin shear bond strength after active application (compared to passive application) was most pronounced for the SE mode. This finding was explained by the fact that rubbing the adhesive in SE mode dissolved the smear layer better than passive application of the adhesive. Due to an increased dissolution of the smear layer, the resin monomers penetrate dentin more readily. Moreover, rubbing the adhesive may accelerate solvent evaporation and may cause a higher amount of monomer to diffuse into the smear layer. Similarly, using CUBQ in SE mode, Seitoku et al^50^ observed insufficient smear-layer removal on the intertubular dentin surface and smear plugs in the dentin tubules during SEM observation. They also proposed that a rubbing motion with longer application time of the ultra-mild adhesive (pH=2.3) may be better, in order to more effectively remove the smear layer. In the latter two in-vitro studies,^4,50^ the dentin was prepared with 600-grit silicon carbide paper. It is important to note that in a clinical situation, a diamond bur is often used to prepare the cavity, creating a thicker smear layer which can interfere more with the penetration of the resin monomers. In the in-vitro study reported by Ahmed et al,^1^ dentin was prepared with a medium-grit (105-µm) diamond bur. They also noticed an improved immediate microtensile bond strength to dentin after a 20-s application of CUBQ (SE and ER mode). However, after aging, a difference was no longer detected between CUBQ-immediate application and CUBQ-application for 20 s. More in-vitro studies with similar study design are required to evaluate the effect of a longer application time of CUBQ on the dentin bond strength after aging.

An evaluation of the bonding effectiveness of CUBQ applied in an SEE mode was preferred over an SE mode in the present study, as several in-vitro studies have demonstrated that selective etching of enamel with phosphoric acid prior to the application of CUBQ improves the bond strength to enamel significantly.^12,18,19,24^ This is a well-known phenomenon for universal adhesives. Indeed, a systematic review evaluating the influence of the application mode of universal adhesives on the clinical performance of NCCL restorations concluded that the ER and SEE application modes showed higher retention rates and less marginal discoloration compared to the SE mode.^26^

FDI criteria were used for the evaluation of the clinical performance of the cervical composite restorations. Since their introduction in 2007, these FDI criteria are increasingly employed in clinical trials.^33^ Very recenty, revised FDI criteria have been published.^20,34^ In this study, only the criteria that were considered clinically relevant for the bonding efficiency of NCCL restorations were included: marginal staining, retention and fracture rate, marginal adaptation, postoperative sensitivity and presence of caries/erosion/abrasion/abfractions. For the marginal staining and marginal adaptation parameters, a further distinction was made between the incisal enamel side and the cervical dentin side in order to evaluate the bonding effectiveness separately at the enamel side and dentin side. This subdivision in marginal integrity at the cervical dentin side and incisal enamel side was used in all previous in-house NCCL clinical trials.^41,42,44,45^

Loss of retention is the main reason for failure in this study. The retention rate was 87.2% for the ER mode and 86.3% for the SEE mode, with no significant difference between both application modes (p > 0.05) (Table 6). The retention rates in this study are much lower in comparison with the retention rates of CUBQ in a 2-year clinical trial of Oz et al.^38^ In this latter study, no restorations were lost when CUBQ was applied following an ER and SEE mode, while the restorations placed with an SE application mode showed a 2-year retention rate of 87.5%.

Looking at the retention rate in 3-year clinical trials evaluating other universal adhesives, loss of retention was in general slightly higher when the universal adhesive was applied in an SE mode. Several clinical trials evaluated Scotchbond Universal (3M Oral Care; St Paul, MN, USA), the first universal adhesive available on the market, after 36 months of clinical functioning.^5,32,39^ Retention rates were recorded of 86%,^39^ 89%,^32^ and 100%^5^ following an SE application mode; 98% for the SEE application mode;^5,32^ and 100%^39^ and 98%^5,32^ for the ER application mode. Gomez de Albuquerque et al^16^ noticed a 3-year retention rate of 87%, 94%, and 93% for FuturaBond Universal (Voco, Cuxhaven; Germany) applied in an SE, SEE and ER mode, respectively. Similar to the present study, the above-mentioned 3-year clinical trials did not show a significant difference in retention rate between the ER and SEE application modes.

Marginal deterioration of the restorations was clearly noticed at the 3-year recall. Only five restorations (3 CUBQ-ER, 2 CUBQ-SEE) showed an unacceptable marginal defect. About 65% of the restorations in each group presented with a slight marginal defect on the enamel and/or dentin side (Table 5). According to the FDI criteria, these slight marginal defects are clinically acceptable (FDI scores 2 and 3). About 55% of the restorations in each group (ER and SEE) had a minor marginal defect (FDI score 2) that can be resolved with refinishing and repolishing of the restoration and is considered clinically irrelevant. In the study by Oz et al,^38^ the percentage of CUBQ-ER and SEE restorations with a clinically acceptable marginal defect was much lower than in our study. In that study, USPHS criteria were used, that have less discriminative power compared to the FDI criteria.^13,20,33,34^

The percentage of clinically acceptable marginal defects in the present study is also higher than in 2-to 3-year clinical trials of NCCLs where other universal adhesives were applied in an ER or SEE mode and FDI evaluation criteria were used.^10,16,32,68^ Two main explanations can be given for this observation. First, evaluation of marginal adaptation is not a completely objective evaluation, especially when dealing with restorations with a clinically good marginal adaptation or a marginal defect that is removable by polishing and finishing (FDI score 2). Second, the bond strength of CUBQ onto enamel and dentin and the Knoop hardness of this adhesive in the early stage (5 min. after polymerization) is significantly lower than after 24 h.^24,65^ Increased polymerization of the adhesive with time goes together with an increased hardness and increased mechanical properties. In addition, the thickness of the adhesive also determines the mechanical properties. The adhesive layer of CUBQ with a thickness of about 10 µm has lower mechanical properties compared to the 2-step SE adhesive Clearfil SE Bond 2 (Kuraray Noritake).^65^ Taking these observations into account, finishing and polishing procedures immediately after composite restoration placement can generate external forces on the restorations in this critical stage, resulting in increased interfacial gap formation.

Regarding the location of the marginal defect on either the incisal enamel or cervical dentin side, there was no significant difference in the percentage of restorations between the two application modes (p > 0.05) (Table 6). This result is expected at the enamel side, as the enamel was etched with phosphoric acid in both groups. At the dentin side, more marginal deterioration would be expected in the ER group compared to the SEE group, following reports in several in-vitro studies that the dentin bond strength of CUBQ used in ER mode is less stable with time compared to the SE mode.^3,6,11,23,25^ However, this was not observed in the present study after 3 years of clinical functioning.

Regarding marginal staining, about 30% of the restorations showed clinically acceptable marginal staining on the incisal enamel and/or the cervical dentin side (Table 5). Clinically still-acceptable marginal staining was almost always associated with the presence of a small but clinically acceptable marginal defect, as observed in many other NCCL clinical trials.^28,35,45,58^ Indeed, small marginal defects create retention places for colorants originating from smoking and dietary habits of patients.

The 3-year clinical success rate in our study was 82.6% for CUBQ-ER and 83.8% for CUBQ-SEE, showing an acceptable clinical performance of the restorations. All lost restorations (16 CUBQ-SEE, 14 CUBQ-ER) needed replacement. The other failed restorations were attributed to the occurrence of a chip fracture at the margin, a severe marginal defect and/or marginal discoloration (3 CUBQ-SEE; 5 CUBQ- ER), but were reparable. The success rates in the present study were lower than in previous in-house 3-year clinical trials evaluating multi-step adhesives such as Optibond FL (Kerr), Permaquick (Ultradent; South Jordan, UT, USA), and Clearfil SE Bond (Kuraray Noritake), or 1-step self-etch adhesives applied without a quick bonding protocol, eg, G-Bond (GC; Tokyo, Japan) and Clearfil S3 Bond (Kuraray Noritake).^35,41,42,45^ These in-house clinical trials showed 3-year success rates between 92% and 100%. From this, we can conclude that strong simplification of the adhesive protocol, for instance, application and direct light curing without waiting (quick/rapid bonding technology), may be more of a marketing advantage than a true benefit. The same conclusion was drawn from other in-vitro studies, showing a significant increase in shear bond strength of CUBQ (ER and SE mode) to dentin and enamel after a double-layer application of the adhesive.^24,65^

Regarding the two application methods (ER and SEE), no significant difference was recorded for any of the parameters evaluated (Table 6). Therefore, the null hypothesis, that there is no difference in clinical effectiveness between both application modes, can be accepted. A similar conclusion was drawn in the 2-year clinical study by Oz et al.^38^

To obtain a sufficiently large sample size of restorations and thus to enhance the power of the study, all cervical lesions per patient were treated and an appropriate statistical method (2-way GEE model) was applied to take the clustered measurements into account.^59^ For each patient, half of the lesions were treated with CUBQ-ER and half with CUBQ-SEE. This is in contrast to most clinical trials evaluating the bonding effectiveness of adhesives in NCCLs, where only one restoration per adhesive or application mode is placed in each patient. As no significant differences were observed for any of the parameters when placing multiple restorations per patient in the present study, it is not likely that the situation would have been different if only two restorations were placed per patient.

The influence of secondary parameters on the clinical outcome of Class-V restorations was also evaluated using a 2-way GEE statistical analysis (Tables 7 and 8). Regarding the operator, the four operators in this study were specially-instructed restorative dentists from the university dental school with a maximum of 3 years of clinical experience. Although the operators in this study were calibrated to perform the restorative procedures, inter-operator differences cannot be ruled out.^9,45,49^ At 3 years, operator 1 had the largest number of failures (13/38), while the lowest number of failures were recorded for operator 4 (12/90). The difference in failure rate between the operators was, however, not significant. In addition, for each operator, the same amount of failures was observed in the ER and SEE group (Table 8).

Regarding the other secondary parameters (height, depth of lesion, presence of wear facets and antagonist, degree of sclerosis), a significant influence was found only for the degree of sclerosis. Pre-operatively, the degree of sclerotic dentin was measured according to the criteria described by Swift et al^52^ (Table 4). For both application methods (ER, SEE), the lowest number of failures was seen in the group of restored teeth without sclerosis (score 1). This result can be expected, as sclerotic dentin is a more difficult substrate to bond to than normal dentin.^55,30^ Sclerotic dentin has partially or totally obliterated dentinal tubules as a result of the continuous deposition of peritubular dentin.^8,14^ The micromorphological features of this altered dentin substrate are potential obstacles to resin infiltration, which include the hypermineralized surface layer, an additional partially mineralized surface bacterial layer, and intratubular mineral casts that are comparatively more acid resistant.^30,31,61^ In-vitro studies have demonstrated that for ER adhesives and self-etch adhesives, bond strengths in sclerotic dentin are 25%-40% lower than those achieved in sound dentin as a result of the presence of an acid-resistant hypermineralized surface layer.^27,54,55,57,64^ In addition, several clinical trials noticed a higher failure rate when restorations were bonded in sclerotic NCCLs using a 1-step SE adhesiveor a universal adhesive.^7,44,45^

## Conclusion

At the 3-year recall, the clinical performance of CUBQ did not depend on the bonding strategy employed (ER or SEE mode). The ease of use of CUBQ, applied following a quick bonding concept, did not positively influence the success rate of the NCCL restorations. Longer-term follow-ups are planned to evaluate the bonding effectiveness of CUBQ after medium- and long-term clinical functioning.

## Clinical relevance

The 3-year clinical performance of the CUBQ restorations did not depend on the bonding strategy employed (etch-and-rinse vs self-etch with prior selective etching of enamel).
